# Three-dimensional evaluation of the root resorption of maxillary incisors after the orthodontic traction of bicortically impacted canines: case reports

**DOI:** 10.1186/s40510-019-0267-z

**Published:** 2019-04-01

**Authors:** Luis Ernesto Arriola-Guillén, Yalil Augusto Rodríguez-Cárdenas, Gustavo Armando Ruíz-Mora, Aron Aliaga-Del Castillo, Juan Schilling, Heraldo Luis Dias-Da Silveira

**Affiliations:** 1 Division of Orthodontics, School of Dentistry, Universidad Científica del Sur, Calle Cantuarias 398, Miraflores, Lima Perú; 2 Division of Oral and Maxillofacial Radiology, School of Dentistry, Universidad Científica del Sur, Calle Cantuarias 398, MIraflores, Lima Perú; 3 Division of Oral and Maxillofacial Radiology, Faculty of Dentistry, Universidad Nacional de Colombia, Bogotá D.C., Colombia; 4 Division of Orthodontics, Faculty of Dentistry, Universidad Nacional de Colombia, Bogotá D.C., Colombia; 5 Department of Orthodontics, Bauru Dental School, University of São Paulo, Bauru, São Paulo, Brazil; 6 Division of Oral Radiology, Faculty of Dentistry, Federal University of Rio Grande do Sul, Porto Alegre, Brazil

**Keywords:** Root resorptions, Canine tooth, Cone-beam CT

## Abstract

**Background:**

The root resorption of the maxillary incisors after the orthodontic traction of impacted canines is a concern for clinicians. The aim of this case series report was to evaluate the root resorption of the maxillary incisors after traction until the occlusal plane of the bicortically impacted canines (placed between the two cortical bones in the middle of the alveolar process) located in a complex position using three-dimensional superimposition. This case series report describes the root resorption of the maxillary incisors after orthodontic traction with NiTi closed coil springs and a heavy anchorage appliance in three cases of bilateral impacted canines located in a complex position (bicortically) near to midline. Cone-beam computed tomographies (CBCTs) were obtained before and after traction. Root resorption in all root surfaces of the maxillary incisors was evaluated with color-coded maps using the ITK-SNAP and the 3D Slicer software to indicate loss of the root surface (in red) or gain of the surface (in blue) and was quantified in millimeters by the superimposition method.

**Results:**

The root changes mainly occurred in the apical third of the maxillary incisor root and did not exceed 2 mm.

**Conclusions:**

Root resorption of the maxillary incisors after the traction of bicortically impacted canines located in a complex position was observed mainly in the apex region, and the amount of root resorption was smaller than 2 mm in all root surfaces.

## Background

Typically, the location of impacted canines is classified into two categories, i.e., a buccal or palatal impacted canine [1–6]. However, in a smaller percentage (approximately 6.6%) of cases, the canines may be impacted in the middle of the alveolar process [7] or precisely between the two cortical bones (bicortical) and cannot be classified as a buccal or palatal canine [8, 9]. These bicortically impacted canines, when located in sector 4 or 5, i.e., near the midline, according to the Ericson and Kurol classification [10] constitute a greater risk for root resorption of the maxillary incisors due to their direct contact.

Orthodontic traction of bicortically impacted canines is considered a highly complex orthodontic treatment due to their direct contact with the root surfaces of the maxillary incisors. The root resorption of the maxillary incisors prior to orthodontic treatment can be observed in some cases with impacted canines [11] but is more frequent in this type of impaction because of its unfavorable eruption trajectory compared to that of buccal or palatal impactions [7]. This phenomenon can increase the risk of root resorption when orthodontic disimpaction is performed due to contact between the root of the maxillary incisor and the crown of the impacted canine [12]. Although the prognosis of these maxillary incisors is reserved, keeping them in the mouth may be preferred to preserve the alveolar bone ridge, especially in younger patients [13–16].

Root resorption of the maxillary incisors has been evaluated mainly on radiographs and using scoring systems. Length, area, and volume assessments have been reported by only a few studies using CBCT [17–19]. This method allows the determination of changes to the structures surrounding the impacted canine, including the resorption produced in the incisor root. Nevertheless, root resorption information has not been presented using three-dimensional superimposition, and therefore, estimating and visualizing the three-dimensional changes produced by canine traction and detecting their locations would be interesting, especially for complex impacted canines [18, 19]. The American Academy of Oral and Maxillofacial Radiology, based on the ALARA principle and the recommendations for the proper use of ionizing radiation, supports the use of cone-beam computed tomography (CBCT) to evaluate impacted canines before and during orthodontic treatment and to control some of the observed negative effects [20, 21].

Methods that allow three-dimensional superimpositions of craniofacial structures have been widely studied [22–27], and their use has increased in recent years since they permit quantitative and qualitative evaluation of the changes produced by growth or by different treatment approaches [24–28]. Among the different analyses that can be performed with three-dimensional superimpositions, color-coded maps permit an interactive visual analytic evaluation of surface displacements [22, 24, 27–29]. These maps can be applied to evaluate root resorption after the orthodontic traction of impacted canines. Thus, the purpose of this case series report was to evaluate the root resorption of maxillary incisors after the traction of bicortically impacted canines located in a complex position through the use of three-dimensional superimposition and color-coded surface maps.

## Materials and methods

This case series report included three patients diagnosed with bilateral canine impaction, including five bicortically maxillary impacted canines and one buccal impacted canine. The patients or their parents, when necessary, provided informed consent before treatment. All cases were treated by one well-trained orthodontist (G.A.R.M) in his private practice from Bogotá, Colombia.

The impacted canines were initially diagnosed using panoramic radiographs. Then, CBCTs were used to carefully study the cases. Canine impaction was evaluated in the sagittal, coronal, and axial sections. The impaction sector according to the Ericson and Kurol classification was evaluated in the sagittal section [10]. The *α* and *β* angles and the impaction height in millimeters were evaluated in the coronal section as diagnostic criteria. The location of the impacted canines (bucco-lingual position) was evaluated in the axial section to assess the position of the crown relative to both cortical bones. The characteristics of the impacted canines in the three patients are described in Table 1.

**Table 1:** Initial characteristics of the patients

Patient characteristics				Impacted canine characteristics					Skeletal characteristics				
Cases	Gender	Age (years)	Angle malocclusion	Impacted side	Impaction sector	*α* angle	*β* angle	Height of impaction	ANB	APDI	SNA	SNB	PNS-ANS
Case 1	Female	19.1	Class I	Right	Sector 5	62.20	40.30	14.30	3.69	83.89	90.81	87.12	54.20
				Left	Sector 5	52.10	28.50	12.60					
Case 2	Male	36.4	Class I	Right	Sector 4	44.80	48.30	9.30	1.88	93.63	91.15	89.27	56.12
				Left	Sector 5	46.90	40.50	10.40					
Case 3	Female	13.3	Class I	Right	Sector 3	48.90	53.40	10.90	3.84	76.27	79.08	75.24	41.68
				Left	Sector 2	22.00	41.20	9.00					

**Fig. 1:**
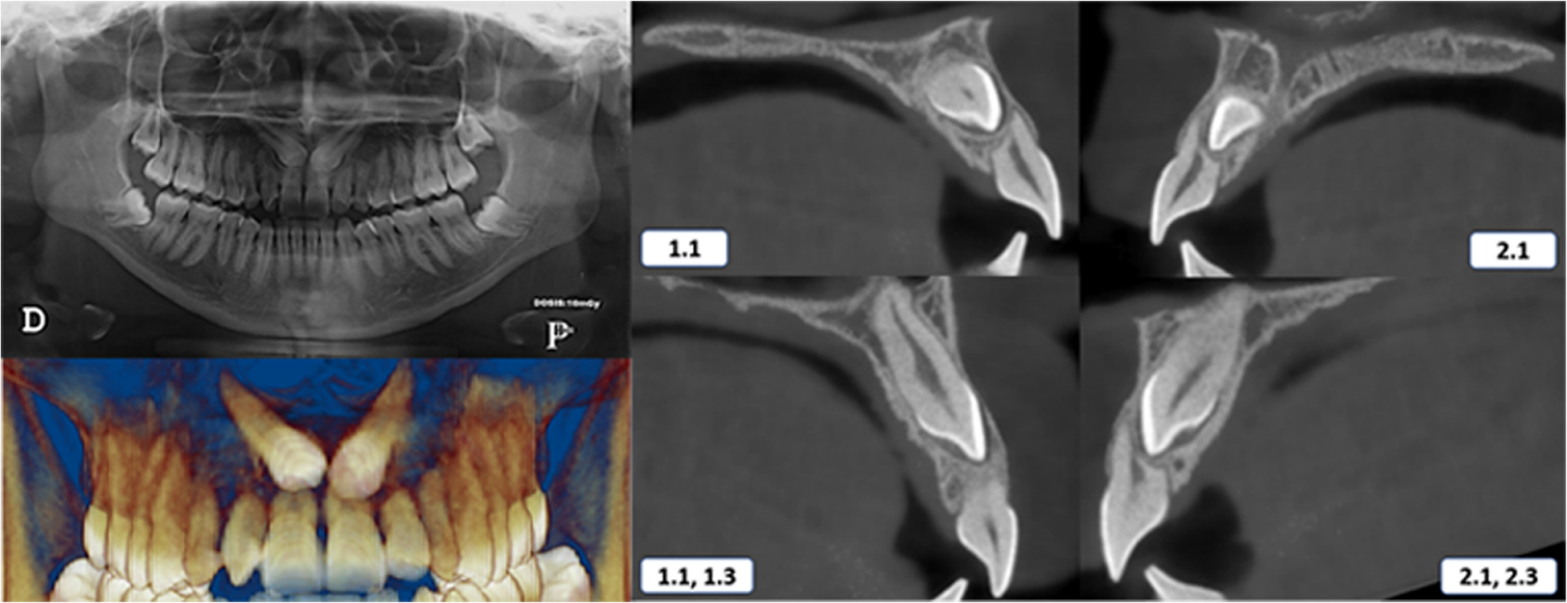
Initial panoramic radiography and CBCT scans—case 1. 1.1, maxillary right central incisor; 1.3, maxillary right canine; 2.1, maxillary left central incisor; 2.3, maxillary left canine

**Fig. 2:**
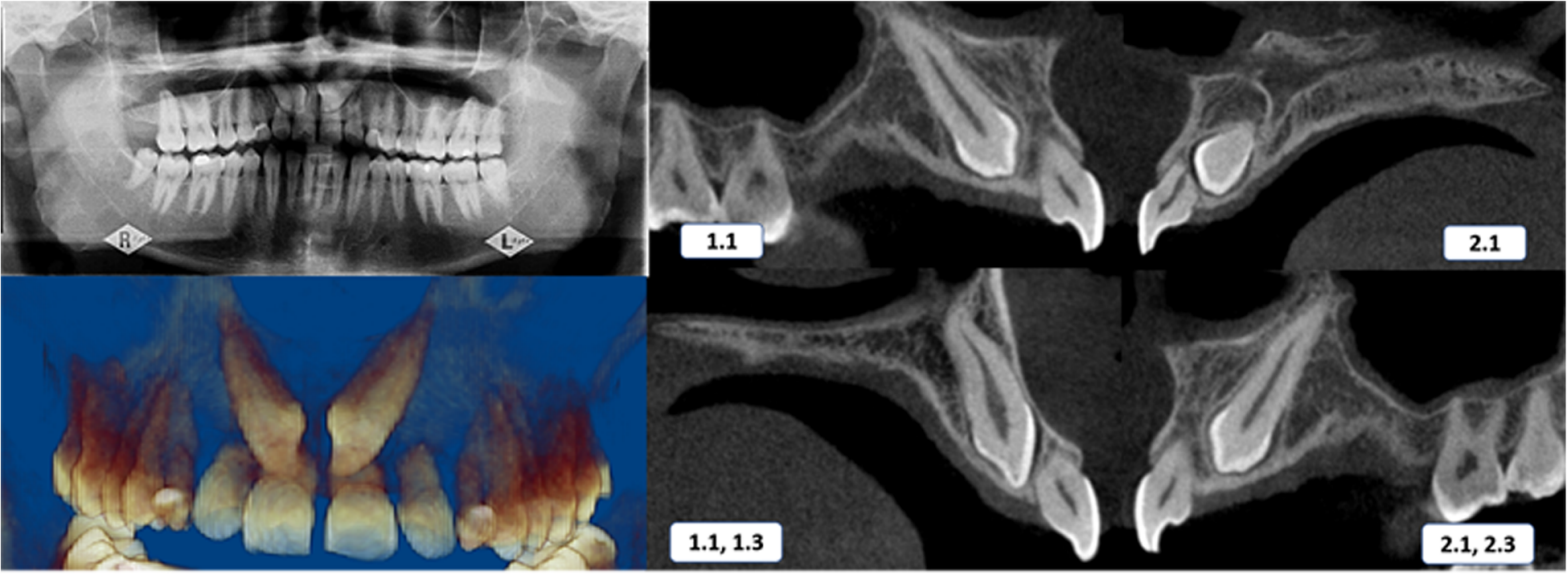
Initial panoramic radiography and CBCT scans—case 2. 1.1, maxillary right central incisor; 1.3, maxillary right canine; 2.1, maxillary left central incisor; 2.3, maxillary left canine

Case 1 was a 19-year-old female with an Angle class I malocclusion and a class I skeletal relationship. The impaction sector on both sides was defined as sector 5 according to the Ericson and Kurol classification [10], and both impacted canines were bicortically located. The right canine had an *α* angle of 62.20° at 14.3 mm from the incisal plane, which caused severe resorption of the roots of the central and lateral incisors. The left canine had an *α* angle of 52.10° at 12.6 mm of the incisal plane (Table 1) (Fig. 1). Case 2 was a 36-year-old male with an Angle class I malocclusion and a class I skeletal relationship. The right canine was located in sector 4 with an *α* angle of 44.8° at 9.3 mm from the incisal plane, which caused severe resorption of the roots of the central and lateral incisors. The left canine was placed in sector 5 with an *α* angle of 46.9° at 10.4 mm of the incisal plane and demonstrated resorption on the central and lateral incisors. The locations for both impacted canines were bicortical (Table 1) (Fig. 2).

**Fig. 3:**
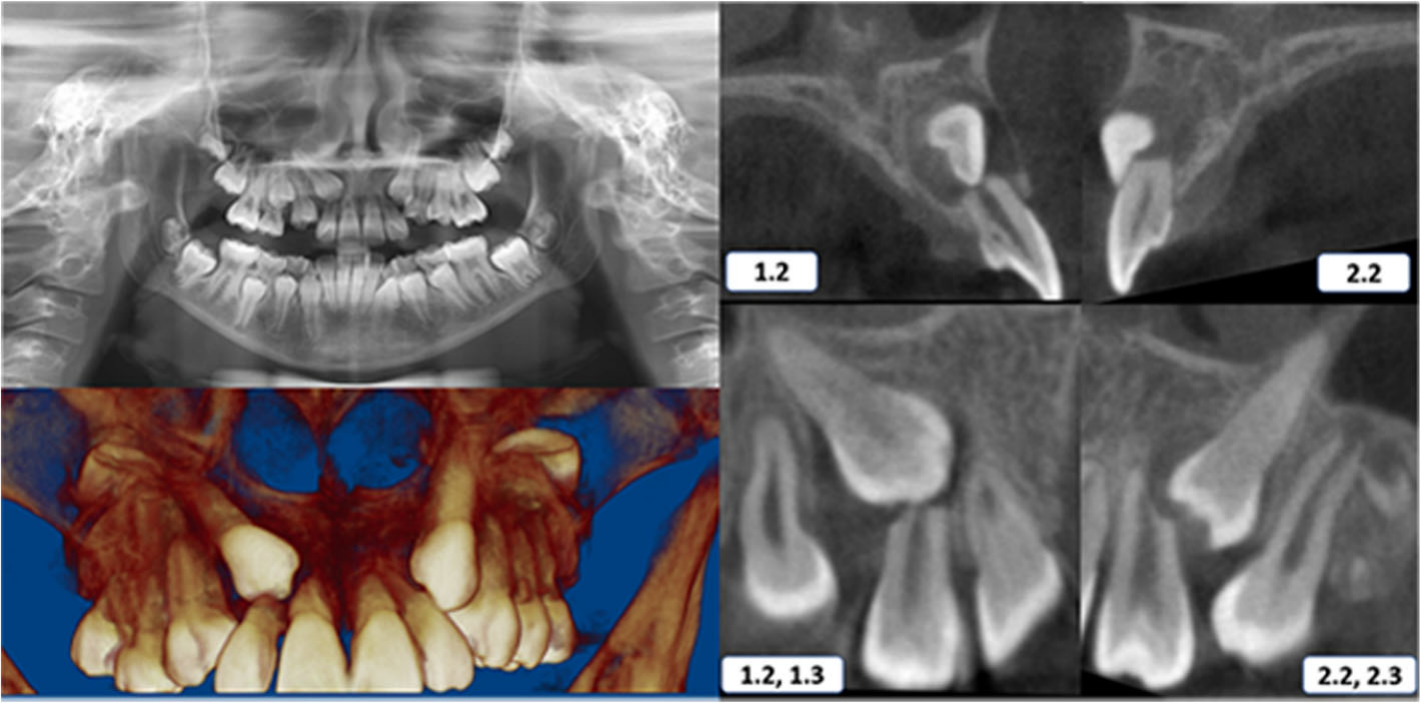
Initial panoramic radiography and CBCT scans—case 3. 1.1, maxillary right central incisor; 1.3, maxillary right canine; 2.1, maxillary left central incisor; 2.3, maxillary left canine

Case 3 was a 13-year-old female with an Angle class I malocclusion and a class I skeletal relationship. The impaction sector on the right side was classified as sector 3 and on the left side was defined as sector 2 according to the Ericson and Kurol classification [10]. The right impacted canine was bicortically located with an *α* angle of 48.9° at 10.9 mm from the incisal plane, which caused severe resorption of the roots, mainly on the lateral incisor. The left impacted canine was located by the buccal side with an *α* angle of 22° at 9 mm from the incisal plane, which caused severe resorption of the roots, mainly on the lateral incisor (Table 1) (Fig. 3).

For the three cases, the main objective was to traction all maxillary impacted canines to the occlusal plane and to avoid greater root resorption of the maxillary incisors to ensure an acceptable dental health status. Thus, to avoid further root resorption, we sought to distance the impacted canine from the roots of the upper incisors. The vectors of the coil springs used to pull the impacted canines in the three cases were the same. The first coil spring pulled the canine in the distal direction and the second coil spring pulled the canine in the occlusal direction until traction was completed. At this moment, the central and lateral incisors were not included in the orthodontic mechanics. Once the impacted canine was separated from the incisor root, these teeth were included in the treatment. Then, the traction mechanics with the coil continued.

**Fig. 4:**
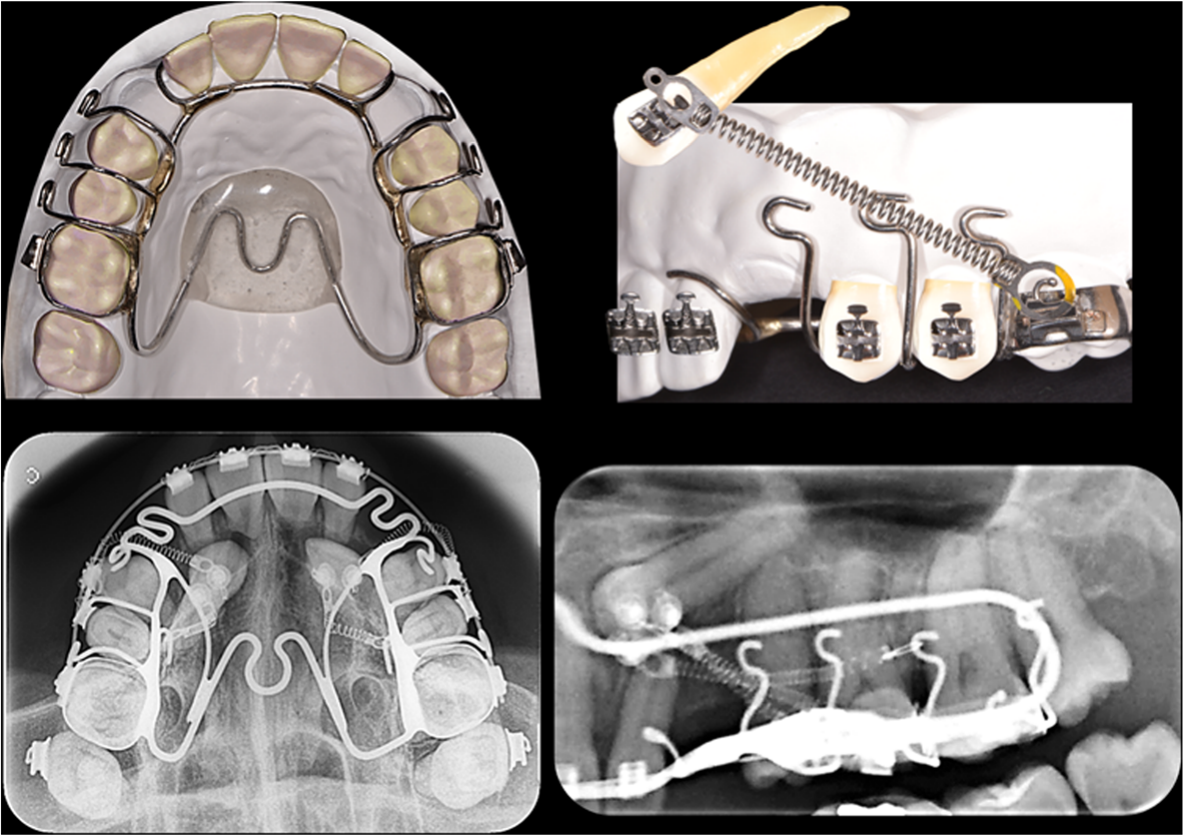
Graphic design and radiographic images of canine traction method

The deciduous canines were extracted when present (cases 1 and 2). All impacted canines were orthodontically tractioned with the same orthodontic mechanics. NiTi closed coil springs and a single rigid heavy reinforced anchorages were used (Fig. 4). The treatment plan for the three cases included fixed orthodontic appliances with 0.022″ × 0.028″ slot metal brackets (Synergy RMO, Inc., Rocky Mountain Orthodontics, Denver, Colorado, USA), and traction of both impacted canines was obtained using NiTi closed coil springs (0.010″ × 0.036″) that were 13 mm and 8 mm in length and had 150 g of force (Dentos Inc. Daegu, Korea) fastened to vestibular hooks in 0.028″ stainless steel wire. These vestibular hooks were welded to the anchorage appliance that included a rigid palatal acrylic button and an arch over the palatal surfaces of all maxillary teeth present in a 1.2-mm (0.047″) stainless steel wire (Dentaurum, GmbH & Co., Ispringen, Germany). All parts of the anchorage appliance were welded in bands that were cemented to the first permanent molars (Fig. 4). The activations were 4 mm to 5 mm (150 g, approximately) every 4 weeks. The canines were tractioned until they reached the occlusal plane.

The CBCT records were obtained at pretreatment (T0) and after the orthodontic traction of the maxillary impacted canines, when the treated canine reached the occlusal plane (T1), to evaluate any undesirable effect of the traction mechanics on the maxillary teeth. All CBCT scans were obtained using the PaX-Uni 3D (Vatech Co., Ltd., Hwaseong, South Korea) with the following parameters: 4.7 mA, 89 KVp, and exposure time of 15 s. Each field of view mode was 8 cm × 8 cm with a voxel size of 0.2 mm.

For the evaluation of root resorption in all root surface of the maxillary incisors, three-dimensional superimposition of the T1 onto the T0 CBCT scans followed by color-coded map evaluation was performed for each incisor as follows.

**Fig. 5:**
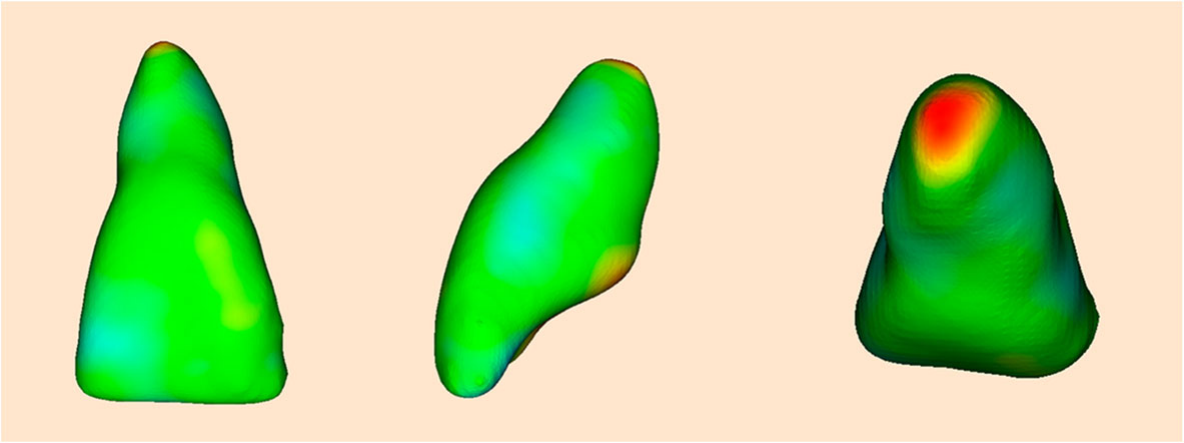
Individual segmentation to create volumetric label maps using the root region at the enamel-cement junction level as the best fit reference

First, the maxillary anterior teeth as a group and then each maxillary incisor individually were segmented from the T0 and T1 CBCT scans to create volumetric label maps by using ITK-SNAP version 2.4 (open source software; www.itksnap.org) (Fig. 5). Then, the virtual three-dimensional surface models for each incisor were created from the T0 and T1 volumetric label maps using the 3D Slicer CMF software (open source software; version 4.0; http://www.slicer.org).

For the three-dimensional superimposition (registration), the T1 scan was registered on the T0 scan, and a fully automated voxel-based registration for each maxillary incisor was performed in the 3D Slicer CMF software using, specifically, the root region at the enamel-cement junction level as the best fit reference [23, 28]. It is important to mention that T1 CBCTs were taken after canine traction [20, 21], when patients were still using brackets. To create T1 models, the brackets were removed from the obtained images due to the presence of artifacts that may influence in the superimposition procedure [30–32]. Therefore, the superimposition was made on the cervical third of the roots and not in the dental crowns.

This software automatically computes and registers the models. Furthermore, the Hounsfield units used to produce the 3D rendered models used a lower threshold of 250 and an upper threshold of 3000; since small variations in the densities viewed in each CBCT DICOM file can affect the rendered results, a manual correction was performed after reviewing each slice of the region of interest to maintain a high-quality model.

After the registration phase, color-coded maps were used to visually analyze the 3D surface displacement (distance) between the two models [33, 34] using the same software. The 3D distances in millimeters between the two surface models at any point of the root surfaces above the root region used for the registration phase could be evaluated [23, 27, 28].

For this specific study, the color-coded surface distance maps have focused only on root displacements between the T0 and T1 models in millimeters. Shades of red represent the root resorption, shades of green or blue indicate no change. Although some change of blue color in the crowns could be seen, this was a consequence of the difficulty to totally remove the streaking artifact of the brackets and does not represent a change in the dental crown.

## Results

**Fig. 6:**
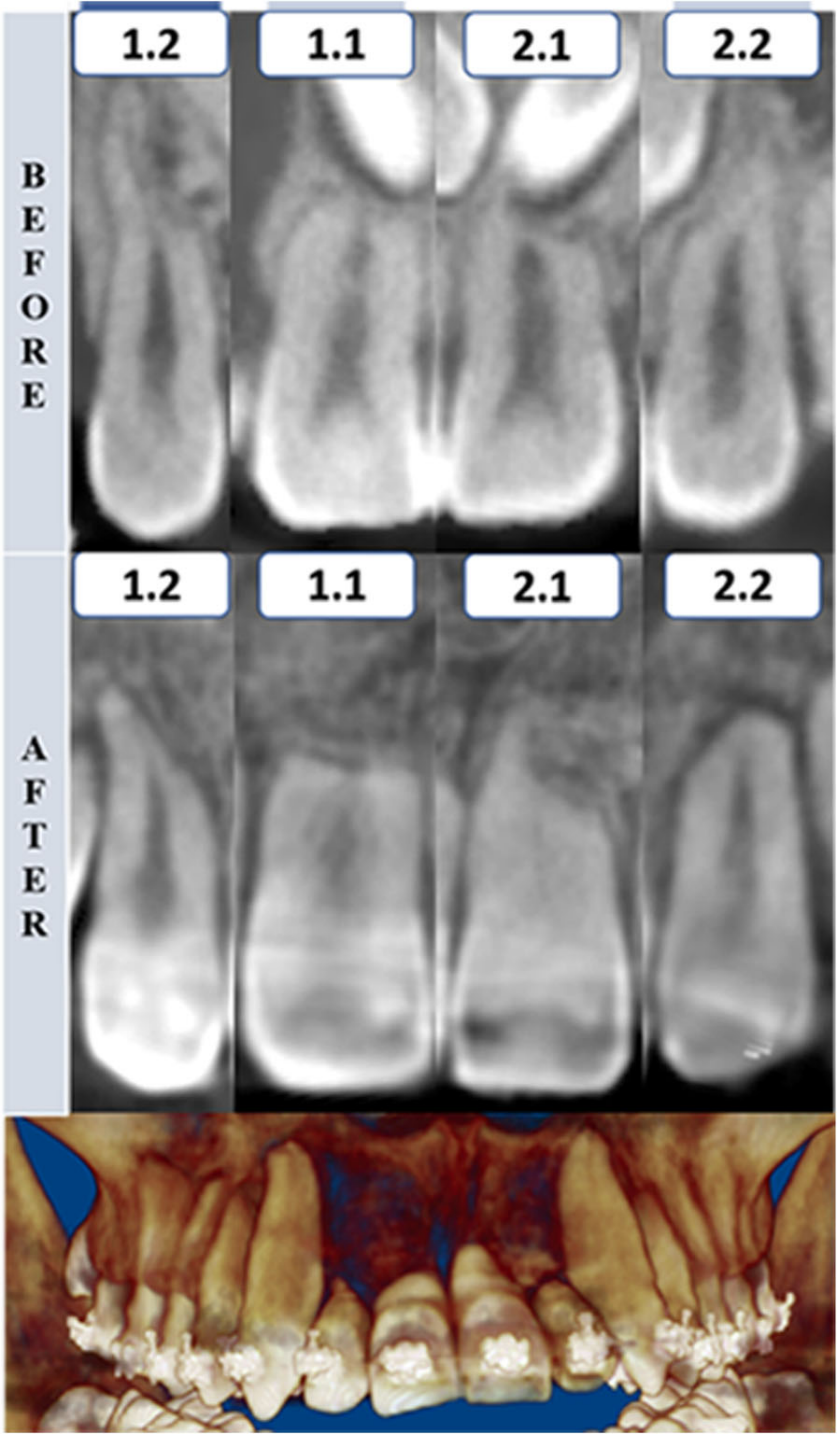
Tomographic rendering after the canine traction and coronal section of maxillary incisors before and after the impacted canine traction—case 1. 1.2, maxillary right lateral incisor; 1.1, maxillary right central incisor; 2.1, maxillary left central incisor; 2.2, maxillary left lateral incisor

**Fig. 7:**
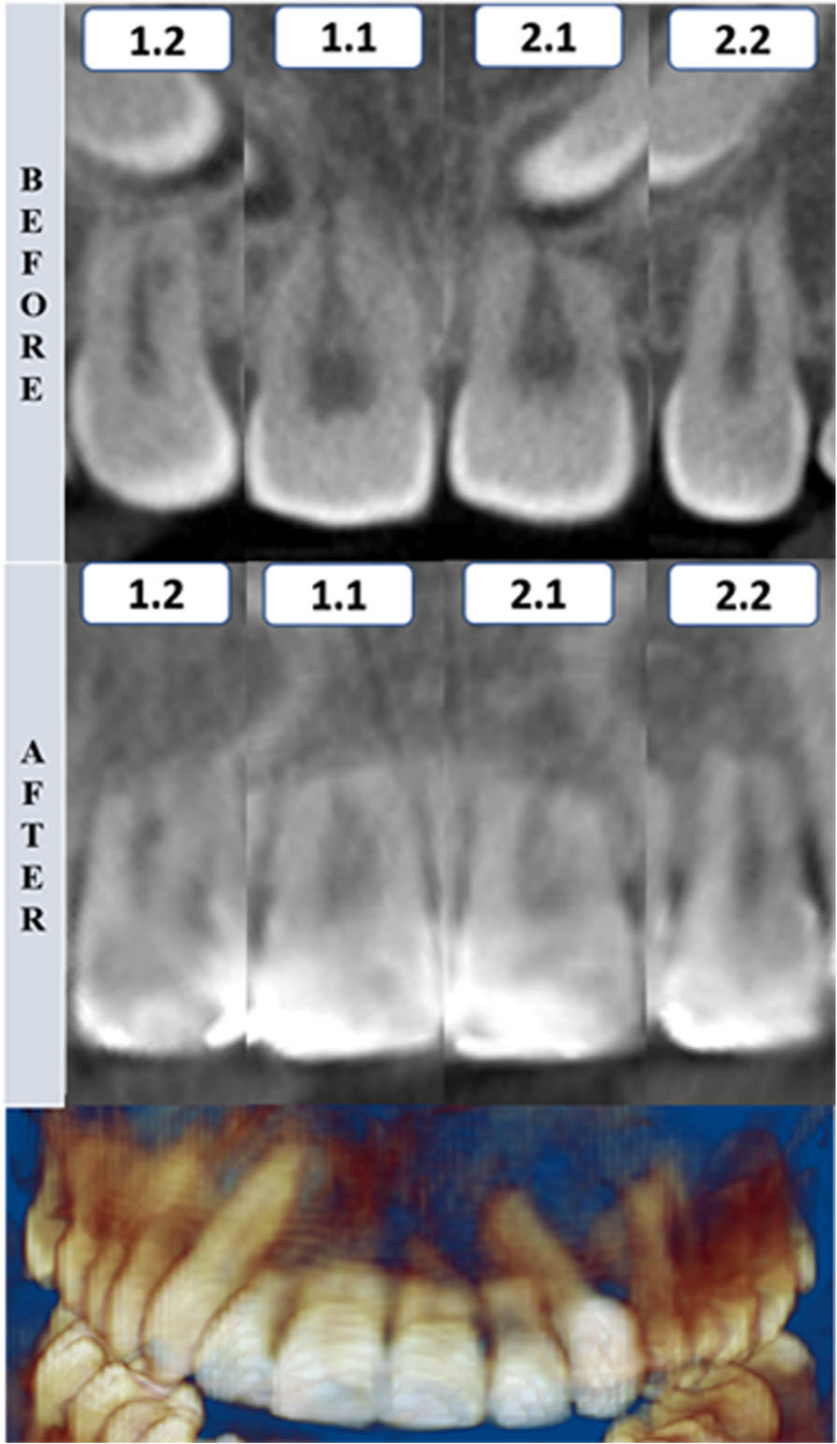
Tomographic rendering after the canine traction and coronal section of maxillary incisors before and after the impacted canine traction—case 2. 1.2, maxillary right lateral incisor; 1.1, maxillary right central incisor; 2.1, maxillary left central incisor; 2.2, maxillary left lateral incisor

**Fig. 8:**
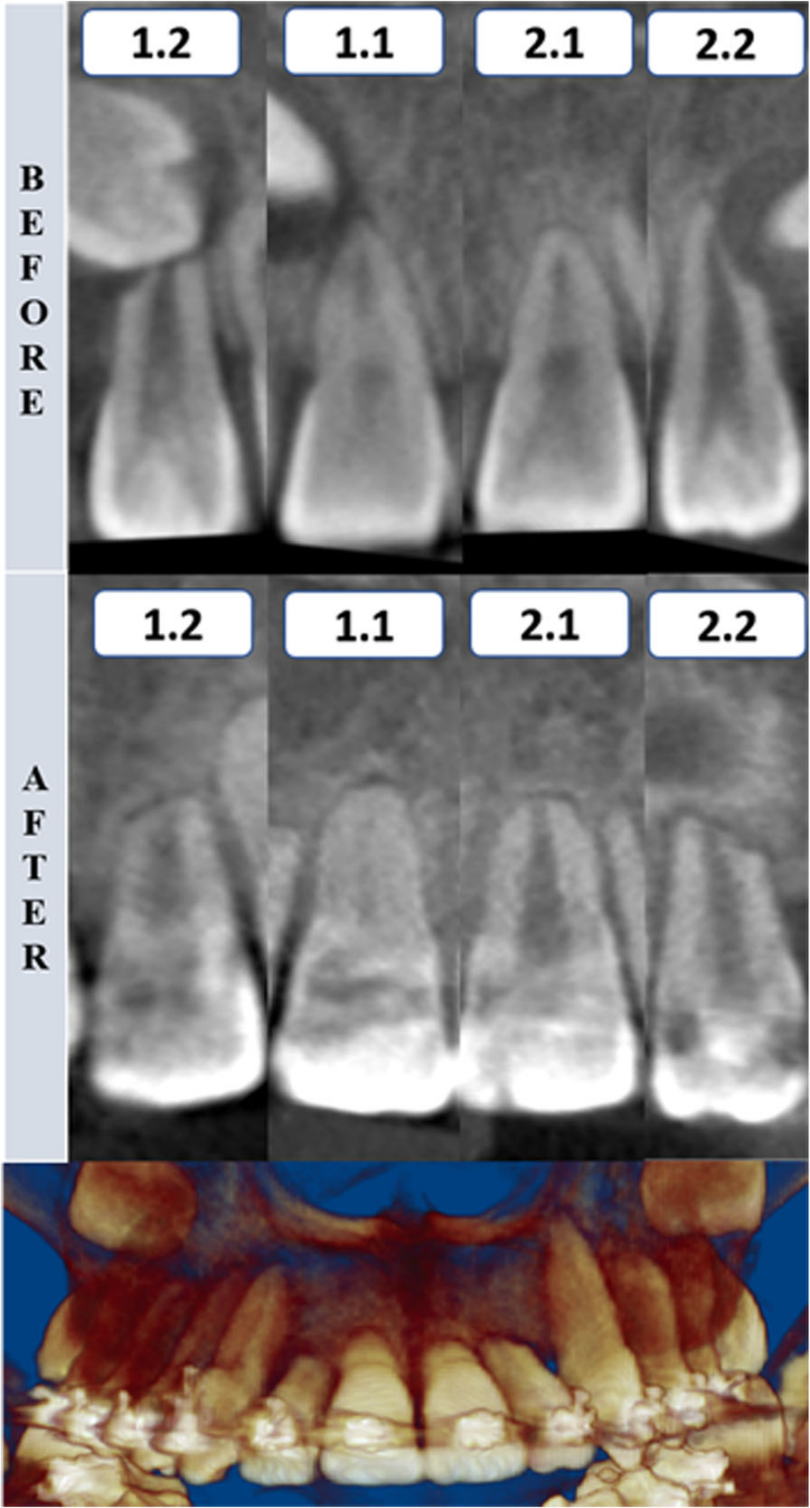
Tomographic rendering after the canine traction and coronal section of maxillary incisors before and after the impacted canine traction—case 3. 1.2, maxillary right lateral incisor; 1.1, maxillary right central incisor; 2.1, maxillary left central incisor; 2.2, maxillary left lateral incisor

The duration of traction in case 1 was 14 months (Fig. 6). In case 2, the duration of traction was 8 months (Fig. 7). Finally, in case 3, the duration of traction was 7 months (Fig. 8). In all three patients, both maxillary impacted canines were tractioned.

The entire three-dimensional superimposition procedure was performed by a calibrated oral radiologist (J.S.) who performed all procedures twice with an interval of 1 month between evaluations.

**Fig. 9:**
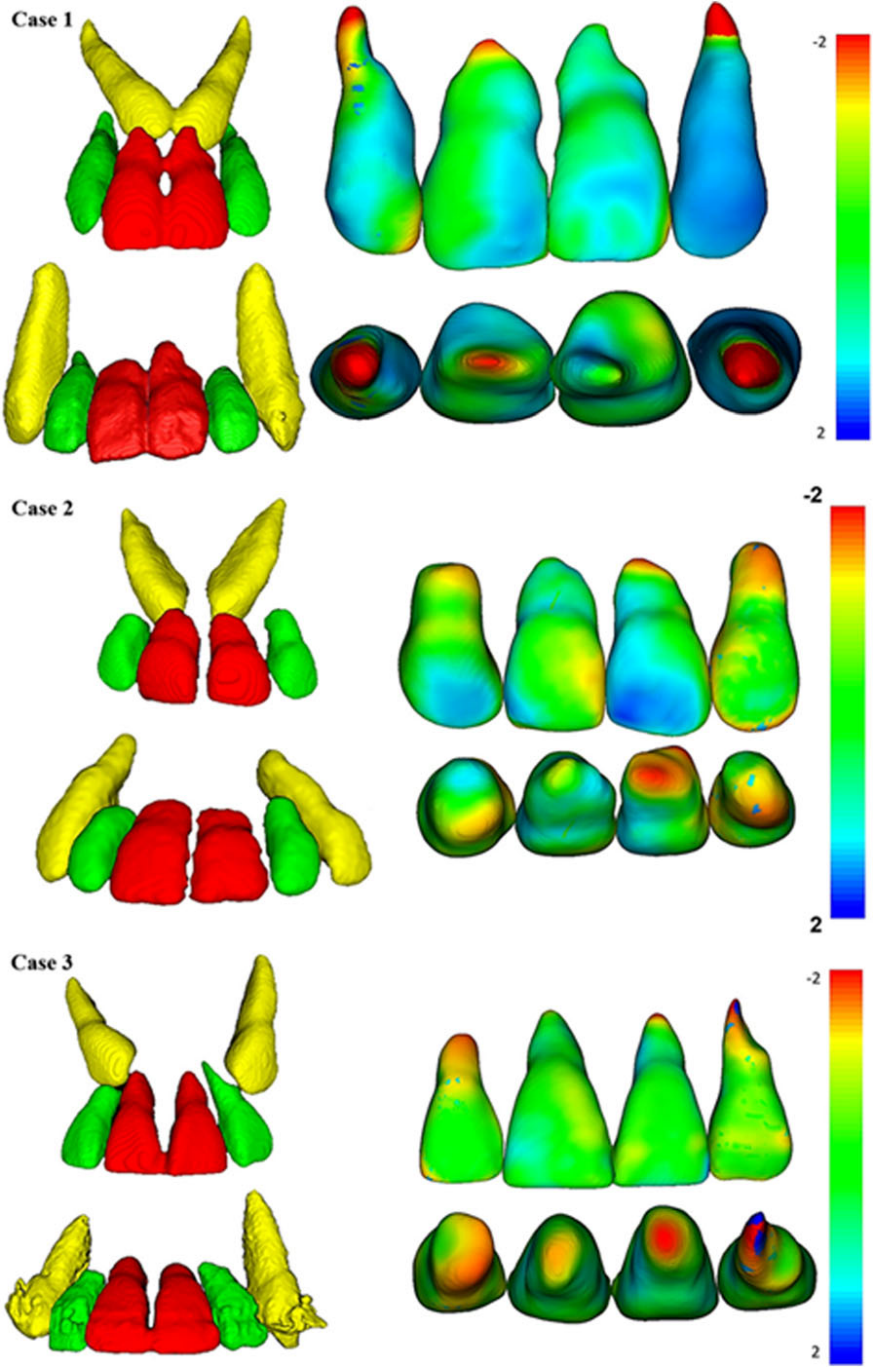
3D superimposition of maxillary incisors: upper figure—case 1 (before and after traction), middle figure—case 2 (before and after traction), and lower figure—Case 3 (before and after traction)

The color-coded surface distance maps showed changes (resorption) mainly in the apical third of the maxillary incisor root, and these changes did not exceed 2 mm (Fig. 9). The red color indicates structure loss.

## Discussion

The purpose of this case series report was to visually quantify the amount of root resorption that occurred after the orthodontic traction of impacted canines until the occlusal plane with an aim of specifically evaluating cases with bicortical canine impaction located very close to the midline. For this analysis, we used color-coded surface distance maps obtained by three-dimensional superimpositions of the initial CBCTs and those taken after canine disimpaction. This evaluation method was described in previous research [22–29]. Although this method has been widely used to evaluate the changes produced by growth or different treatments [24–28], it has not been used to evaluate root resorption after canine disimpaction. Therefore, this case series report makes an effort to introduce this type of three-dimensional analysis to the root resorption evaluation field. The strength of this method is that it allows easy identification of the regions and quantification of the amount of root resorption by visual examination. Some unavoidable imperfections in dental crown surfaces were observed after the segmentation process of T1 CBCTs. This was a consequence of the difficulty on achieving a complete removal of streaking artifact of the brackets from T1 scans [30–32]. Then, they were evidenced in the superimposition, as expected. For this reason and to avoid the crown area imperfections that may affect the superimposition method, the models were registered at the enamel-cement junction level.

The voxel-based image registration method was used to perform the three-dimensional superimposition. This method has been reported to be an accurate and reproducible semiautomated technique for 3D CBCT superimposition, and its use has increased in recent years [22–29, 33, 35–38]. Because the method requires skill and expertise to handle the specific software, all three-dimensional superimpositions were performed by an expert and calibrated radiologist (J.S.), which ensured the reliability of the results.

Bicortically impacted canines (placed between the two cortical bones in the middle of the alveolar process) located close to midline are considered a risk factor for the root resorption of maxillary incisors due to the proximity or direct contact with their roots [7–9]. Therefore, the orthodontic traction of these canines may have some complexity because canine traction can increase the contact between the canine and the incisor root. For this reason, special orthodontic biomechanics should be considered. In this study, the orthodontic traction was performed exclusively by one expert orthodontist with more than 20 years of experience in the treatment of impacted canines (G.A.R.M) to ensure a single traction technique and the efficiency of the treatments.

The cases presented in this study had complex impacted canines characterized by their location, type of impaction, and large amount of initial root resorption in at least one maxillary incisor. Therefore, a special traction method was necessary. The orthodontic treatment included three specific characteristics: the use of a heavy orthodontic reinforced anchorage (1.2″ stainless steel wire) [17], the use of continuous tensile forces produced by the NiTi closed coil springs, and the use of wire extensions (hooks) derived from the anchor unit that allowed control of the traction direction and prevented contact of the coil springs with the gingiva. The purpose of this treatment protocol was to avoid any undesirable effect on the maxillary incisors.

Despite the difficulty in orthodontic traction of maxillary impacted canines, the amount of root resorption of the maxillary incisors in these cases was clinically acceptable. The root resorption was mainly located in the apical region, and no incisor showed root resorption greater than 2 mm. An important characteristic observed in these patients was the irregular morphology of the maxillary incisor roots at pretreatment, with some regions showing considerable root resorption. These regions were the areas in which root resorption was evident after traction. Again, these root resorptions were mainly observed at the apical third. Likewise, no root resorption was observed in the middle or cervical thirds, as shown in the color-coded maps of all three-dimensional superimpositions.

This study aimed to evaluate the root resorption of incisors after the completion of traction of the impacted canines to the occlusal plane, which is a critical phase of orthodontic treatment for this type of malocclusions due to the greater risk of contacting the canine with the incisor roots, as mentioned above. Although root resorption can be expected to increase until the end of the comprehensive orthodontic treatment, this increase may not be clinically relevant due to the short remaining treatment time. However, this issue should be further evaluated in future studies. Nevertheless, the acquisition of a control CBCT after treatment should be well justified [21].

This study is one of the first reports of the use of this method for the evaluation of root changes after canine traction, including patients with complex canine impactions. Studies with considerable sample sizes and adequate designs should be performed. Another important consideration is that the majority of the patients presented alveolar bone around the incisor roots. This phenomenon was observed in the CBCT scans after orthodontic traction. This condition was favorable and generated a good prognosis.

Although the cases presented root resorption before treatment, this resorption was not a contraindication for canine traction. An argument could be made that patients showing initial resorption of the maxillary incisors should not be included in the treatment. However, these patients presented good alveolar bone condition. Moreover, since the majority of the patients were young, keeping the incisors in the mouth was considered important to preserve the alveolar bone ridge in the anterior region. Nevertheless, the stability of these maxillary incisors should be further evaluated with long-term follow-up records.

## Conclusion

For this case series report, the color-coded surface distance maps obtained by three-dimensional superimpositions showed that the amount of root resorption of the maxillary incisors after the traction of bicortically impacted canines was located mainly in the apex region and was smaller than 2 mm in all root surfaces.

## Abbreviations

3D: Three-dimensional
ALARA: Principle: radiation dose “As Low As Reasonably Achievable”
CBCT: Cone-beam computed tomography
